# Supplementary material for: Stacking Model‐Based Classifiers for Dealing With Multiple Sets of Noisy Labels
Source: Biom J. 2025 Mar 12;67(2):e70042. doi: 10.1002/bimj.70042 (PMC11898607; doi:10.1002/bimj.70042)
Supplement: Supplementary file 1 — Supporting Information [file BIMJ-67-e70042-s001.zip › density-based-ensemble-model-main/competitors_real_data/netlab-master/netlab-master/help/mlptrain.htm]

Netlab Reference Manual mlptrain


# mlptrain

## Purpose

Utility to train an MLP network for demtrain

## Description

`[net, error] = mlptrain(net, x, t, its)` trains a network data
structure `net` using the scaled conjugate gradient algorithm
for `its` cycles with
input data `x`, target data `t`.

## See Also

`demtrain`, `scg`, `netopt`

---

**Pages:**
Index


---

Copyright (c) Ian T Nabney (1996-9)
